# Muscarinic Modulation of Network Excitability and Short-Term Dynamics in the Dorsal and Ventral Hippocampus.

**DOI:** 10.17912/micropub.biology.001367

**Published:** 2024-12-19

**Authors:** Giota Tsotsokou, Milena Fassea, Costas Papatheodoropoulos

**Affiliations:** 1 Laboratory of Physiology, Department of Medicine, University of Patras, Pátrai, West Greece, Greece

## Abstract

Cholinergic transmission fundamentally modulates information processing in the brain via muscarinic receptors. Using *in vitro* electrophysiological recordings of population spikes from the CA1 region, we found that the muscarinic receptor agonist carbachol (CCh, 1 μM) enhances the basal excitation level in the dorsal but not ventral hippocampus. Using a frequency stimulation protocol, we found that CCh transforms depression of neuronal output into facilitation (at 3-30 Hz) in the ventral hippocampus while only lessening depression in the dorsal hippocampus, suggesting that muscarinic transmission boosts basal neuronal activation in the dorsal hippocampus and strongly facilitates the output of the ventral hippocampus in a frequency-dependent manner.

**Figure 1. Effects of muscarinic cholinergic transmission on local network excitation and short-term dynamics in dorsal and ventral hippocampus.**
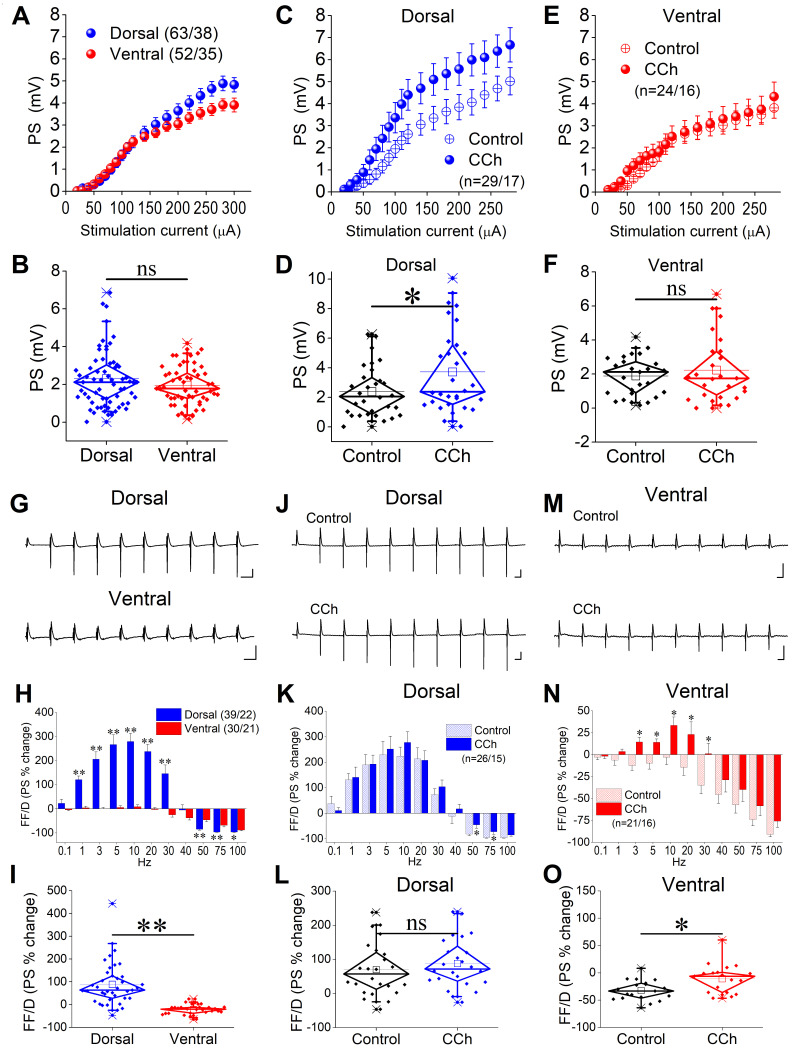
**Figure 1. Effects of muscarinic cholinergic transmission on local network excitation and short-term dynamics in dorsal and ventral hippocampus.** **A** . Input-output relationship between PS and stimulation current intensity are shown for the dorsal and ventral hippocampus under control conditions. **B** . Average values of PS calculated from the corresponding curves in A are shown for the dorsal and ventral hippocampus. **C** & **D** . Input-output curves between PS and stimulation current intensity before (Control) and after application of 1 μM carbachol (CCh) (C) and the corresponding average values of PS calculated from the input-output curves (D) are shown for the dorsal hippocampus. **E** & **F** . Input-output curves between PS and stimulation current intensity before (Control) and after application of 1 μM carbachol (CCh) (E) and the corresponding average values of PS calculated from the input-output curves (F) are shown for the ventral hippocampus. **G** . Examples of PS evoked by a ten-pulse stimulation train (frequency stimulation) at the frequency of 5 Hz obtained from a dorsal and a ventral hippocampal slice. **H** . Cumulative graph of PS changes during frequency stimulation obtained from the dorsal and ventral hippocampus. **I** . Graph showing the average percent effect of frequency stimulation on PS in the two hippocampal segments. **J** . Examples of PS evoked by the frequency stimulation protocol in a dorsal hippocampal slice. **K** . Cumulative graph of PS changes during frequency stimulation obtained before and after application of 1 μM CCh in the dorsal hippocampus. **L** . Average values of percent effect of frequency stimulation on PS before and after CCh application in the dorsal hippocampus. **M** . Examples of PS evoked by the frequency stimulation protocol in a ventral hippocampal slice. **N** . Cumulative graph of PS changes during frequency stimulation obtained before and after application of 1 μM CCh in the ventral hippocampus. **O** . Average values of percent effect of frequency stimulation on PS before and after CCh application in the ventral hippocampus. Average data presented by box and whisker plots in this figure show the median with the 25 ^th^ and 75 ^th^ quartiles (diamond box), the mean and the 5 ^th^ and 95 ^th^ percentile (thick line through small box and whiskers, respectively), and individual data points. Numbers into parenthesis represent the number of slices and animals used (slices/rats). Asterisks denote statistically significant differences at * *p* <0.05 and ** *p* <0.001). “ns” denotes statistically not significant difference. We used the independent t-test for comparisons between the two segments of the hippocampus and the paired t-test for assessing drug effects. We used the Levene’s test to examine population variances for equality.

## Description

Cholinergic inputs from the forebrain regions medial septal area and the diagonal band of Broca deeply modulate the function of hippocampal network (Teles-Grilo Ruivo and Mellor, 2013) and muscarinic cholinergic transmission is crucially involved in hippocampus-dependent behaviours including exploration, learning and memory, synaptic plasticity, and social cognition (Atherton et al., 2015; Bianchi et al., 2003; Dannenberg et al., 2017; Haam and Yakel, 2017; Hasselmo, 2006; Pimpinella et al., 2021) . The actions of acetylcholine are mediated through nicotinic receptors that are non-selective cation channels and G protein-coupled muscarinic receptors (Thiele, 2013) . While nicotinic receptors are exclusively excitatory engaged in point-to-point neurotransmission, muscarinic receptors are excitatory when coupled to G _q/11_ proteins or inhibitory when coupled to G _i/o_ proteins and function to mediate the main neuromodulatory actions of acetylcholine (Brown, 2010; Picciotto et al., 2012; Thiele, 2013; Wess et al., 1996) .

In the brain, muscarinic receptors are expressed both at presynaptic nerve terminals and postsynaptic sites (Picciotto et al., 2012; Thiele, 2013) . In several brain regions, including the hippocampus, prominent muscarinic actions consist of a presynaptic inhibition of neurotransmitter release, and an excitation of postsynaptic neurons (Dasari and Gulledge, 2011; Hasselmo and Schnell, 1994; Thiele, 2013; Thorn et al., 2017) ; by these actions, muscarinic transmission dynamically modulate neuronal activity in brain networks (Alger et al., 2014; Dannenberg et al., 2017; Teles-Grilo Ruivo and Mellor, 2013; Thiele, 2013) .

Recent evidence points to a non-homogeneous pattern of actions of the cholinergic system along the dorsoventral axis of the hippocampus. Notably, it has been shown that nicotinic transmission via α7 receptors preferentially mediate long-term synaptic plasticity in the dorsal but not ventral CA1 hippocampal region (Tsotsokou et al., 2024a), while muscarinic M4 and M2 receptors facilitate short-term synaptic plasticity in the CA1 region more in ventral than dorsal hippocampus (Tsotsokou et al., 2024b). The muscarinic actions on short-term synaptic plasticity along the hippocampus are frequency-dependent (Tsotsokou et al., 2024b), thereby defining the temporal properties of synaptic inputs in the CA1 region. However, although changes in synaptic transmission play an important role in determining the properties of information spread through synaptic circuits, it is the neuronal output of a neuronal network that ultimately determines the communication of a brain region with its target areas and expresses the effect of a neuromodulator on the information flow between brain regions (Silver, 2010) . Hence, in the present study, we sought to explore how the muscarinic cholinergic transmission modulates the frequency-dependent properties of hippocampal output.

First, we found that PS amplitude is similar in dorsal and ventral hippocampus (t _106.9_ = 1.256, *p* =0.197; Fig. 1A -B). Then, we found that 1 μM CCh produced an upward shift in the input-output curve in dorsal ( Fig. 1C -D) but not ventral hippocampal slices ( Fig. 1E -F). Accordingly, CCh significantly enhances the average PS in the dorsal (t _28_ = -2.657, *p* =0.013; Fig. 1D ) but not the ventral hippocampus (t _23_ = -0.701, *p* =0.491) ( Fig. 1F ).

In line with previous reports (Koutsoumpa and Papatheodoropoulos, 2019; Trompoukis et al., 2022) we found that short-term neuronal dynamics (STND) in dorsal and ventral hippocampus display strikingly distinct frequency-dependent profiles ( Fig. 1G,H,I ). Specifically, in the dorsal but not the ventral hippocampus we observed robust frequency facilitation of neuronal output at stimulation frequencies from 1 to 30 Hz ( Fig. 1H ). Furthermore, at higher stimulation frequencies, from 50 to 100 Hz, the neuronal output is depressed in both segments of the hippocampus but to a greater extent in the dorsal than ventral hippocampus ( Fig. 1H ). When the average effect of frequency facilitation was considered, the dorsal hippocampus showed frequency facilitation (FF) while the ventral hippocampus showed frequency depression (FD) (81.85±12.3% and -12.37±7.85%, respectively; t _58.7_ = 6.047, *p* < 0.001) ( Fig. 1I ).

Then, we examined how CCh modulates the output of the CA1 hippocampal region under conditions of repeated activation. We found that 1 μM CCh only reduced the amount of FD in the dorsal hippocampus at relatively high stimulation frequencies (50 and 75 Hz; *p* < 0.05, n=26/15; Fig. 1J -K), with not statistically significant average effect ( *p* > 0.05; left panel in Fig. 1L ). In contrast, application of 1 μM CCh in the ventral hippocampus produced a robust change in STND by transforming FD into FF at the frequency range of 3 - 30 Hz and eliminating FD at 30 Hz (comparison at individual stimulation frequencies, *p* < 0.05, n=21/16; Fig. 1M -N), with a significant average effect ( *p* < 0.05; Fig. 1O ).

Muscarinic cholinergic transmission exerts both excitatory and inhibitory actions by acting through five muscarinic receptor subtypes (Behrends and ten Bruggencate, 1993; Thiele, 2013; Wess, 2004) . Notably, M1-type muscarinic receptors (M1, M3, and M5) mediate mostly excitatory actions, while M2-type muscarinic receptors (M2 and M4) mediate inhibitory actions (Brown, 2010; Dasari and Gulledge, 2011) . M1-type receptor-dependent actions are mostly postsynaptic and are mediated mainly by down regulation of various potassium channels, while M2-type receptor-dependent inhibitory actions involve activation of potassium channels, down regulation of calcium channels, and inhibition of neurotransmitter release (Brown, 2010; Dasari and Gulledge, 2011; Giessel and Sabatini, 2010; McCormick and Prince, 1986; Wickman and Clapham, 1995) . The M1 is the predominant muscarinic receptor subtype in CA1 hippocampal pyramidal cells, while M2 and M4 subtypes are also densely expressed in the CA hippocampal regions (Levey et al., 1995; Rouse et al., 1998; Rouse et al., 1999; Walker and Lawrence, 2020) . In addition to the modulation of principal cell activity, muscarinic receptors regulate the activity of different types of inhibitory interneurons (Behrends and ten Bruggencate, 1993; Pitler and Alger, 1992a, b; Volpicelli and Levey, 2004) . For instance, activation of M1 muscarinic receptors enhances the excitation of parvalbumin-containing neurons in the CA1 hippocampal region (Yi et al., 2014) .

We show that 1 μM CCh enhances PS in the dorsal but not ventral CA1 region. Considering that muscarinic activity modulates both excitatory and inhibitory influences in the hippocampal network (Behrends and ten Bruggencate, 1993; Brown, 2010; Dasari and Gulledge, 2011; Giessel and Sabatini, 2010; McCormick and Prince, 1986; Pitler and Alger, 1992a; Thiele, 2013; Volpicelli and Levey, 2004; Wickman and Clapham, 1995) , and that PS results from the balance between excitation and inhibition in the network, the enhancing effects of CCh on PS in the dorsal hippocampus could be explained by a higher drug effect on the network excitation and/or a relatively minor effect on network inhibition. Likewise, the absence of CCh effects on PS in the ventral hippocampus could be interpreted as the result of balanced drug-induced changes on network excitation and inhibition. Apparently, detailed future research on the muscarinic actions on dorsal and ventral hippocampal principal cells and interneurons is required to clarify the mechanisms of the different dorsoventral effects of muscarinic activation.

In contrast to the higher effect of CCh on PS, CCh affected short-term neuronal dynamics (STND) more in ventral than dorsal hippocampus. Notably, in the ventral hippocampus, CCh reverses frequency depression into frequency facilitation at the stimulation frequency range of 3-30 Hz. Mechanisms that contribute to determining the specific profile of STND include short-term synaptic plasticity and GABAergic inhibition (Koutsoumpa and Papatheodoropoulos, 2019; Leung and Fu, 1994; Margineanu and Wülfert, 2000; Michelson et al., 1989) . A previous study (Koutsoumpa and Papatheodoropoulos, 2019) has shown that GABAergic inhibition modulates STND in dorsal hippocampus at 30-100 Hz, and in the ventral hippocampus at 3-100 Hz which covers the frequencies of muscarinic modulation of STND in this hippocampal segment. Muscarinic transmission mediates suppression of GABA release in the hippocampus through M2 receptors (Levey et al., 1995; Volpicelli and Levey, 2004) . M2 receptors in the hippocampus are expressed mostly in interneurons (Ferraguti et al., 2005; González et al., 2008; Hájos et al., 1998; Katona et al., 2020; Levey et al., 1995; Seeger et al., 2004). Interestingly, M2 receptors display a two-fold higher expression in the ventral compared with the dorsal hippocampus (Tsotsokou et al., 2024b). Accordingly, the present results suggest that muscarinic modulation of STND in the ventral hippocampus might involve a reduction in GABAergic inhibition.

STND represents the temporal properties of the collective output of a local neuronal network under specific conditions, and, therefore, is crucial in determining the mode of communication between brain regions (Haider and McCormick, 2009; Womelsdorf et al., 2014) . Thus, the CCh-induced robust modulation of STND in the ventral but not dorsal hippocampus and the increase in basal network excitation in the dorsal but not ventral hippocampus suggests that muscarinic cholinergic transmission is profoundly involved in modulating the temporal characteristics of the ventral hippocampus output while enhances the output of the dorsal hippocampus in a frequency-independent manner. Considering that the ventral hippocampus is importantly involved in functions such as stress response, anxiety, fear, and social cognition (Hong and Kaang, 2022) by targeting a plethora of other brain regions including medial prefrontal cortex, basolateral amygdala, nucleus accumbens, and hypothalamus (Gergues et al., 2020) , the present data point to a fundamental role of muscarinic transmission in several brain functions via modulation of the temporal dynamics of the ventral hippocampus output.

## Methods

We recorded evoked population spike (PS) from the stratum pyramidale of the CA1 area of dorsal and ventral hippocampal slices obtained from male Wistar rats (RRID:RGD_2312511; originally obtained from CharlesRiver France and currently maintained in the Institutional Laboratory of Experimental Animals) aged 3-4 months. All experimental procedures were conducted in accordance with the European Communities Council Directive Guidelines for the care and use of Laboratory animals (2010/63/EU – European Commission) and approved by the Directorate of Veterinary Services of the Achaia Prefecture of Western Greece Region (reg. number: 187531/626, 26/06/2018), and the Research Ethics Committee of the University of Patras. We prepared and maintained slices in an interface type recording chamber as previously described (Koutsoumpa and Papatheodoropoulos, 2019) . Recordings were performed at 30.0±0.5 ^o^ C in standard medium containing (in mM): 124 NaCl, 4 KCl, 2 CaCl _2_ , 2 MgSO _4_ , 26 NaHCO _3_ , 1.25 NaH _2_ PO _4_ and 10 glucose, and equilibrated with 95% O _2_ and 5% CO _2_ gas mixture at a pH=7.4. PS was quantified by its amplitude. Short-term changes in PS (short-term neuronal dynamics, STND) were studied using a ten-pulse train delivered at varying frequency from 0.1 to 100 Hz (frequency stimulation). The muscarinic agonist 2-Hydroxyethyl)trimethylammonium chloride carbamate (Carbamoylcholine chloride, CCh) was applied at 1 μM for 30 minutes. The values in the text and figures correspond to mean ± S.E.M. Throughout the text the number of slices and animals used in the study is provided (slices/animals).

## Reagents

2-Hydroxyethyl)trimethylammonium chloride carbamate.
